# Herbicide options for effective weed management in dry direct-seeded rice under scented rice-wheat rotation of western Indo-Gangetic Plains

**DOI:** 10.1016/j.cropro.2015.12.021

**Published:** 2016-03

**Authors:** Vijay Singh, Mangi L. Jat, Zahoor A. Ganie, Bhagirath S. Chauhan, Raj K. Gupta

**Affiliations:** a Dept. of Crop, Soil, and Environmental Sciences, University of Arkansas, Fayetteville 72701, AR, USA; b International Maize and Wheat Improvement Center, NASC complex, New Delhi 110012, India; c Dept. of Agronomy and Horticulture, University of Nebraska-Lincoln, NE 68583, USA; d Queensland Alliance for Agriculture and Food Innovation (QAAFI), The University of Queensland, Toowoomba 4350, Australia

**Keywords:** B:C ratio, Crop injury, Dry direct-seeded rice, Herbicides, POST, PRE, Weed management

## Abstract

Farmers' participatory field trials were conducted at Madhuban, and Taraori, the two participatory experimental sites/locations of the Cereal Systems Initiative for South Asia (CSISA), a collaborative project of IRRI and CIMMYT in Karnal district of Haryana, India, during *Kharif* (wet season) 2010 and 2011. This research aimed to evaluate preemergence (PRE) and postemergence (POST) herbicides for providing feasible and economically viable weed management options to farmers for predominant scented rice varieties. Treatments with pendimethalin PRE fb bispyribac-sodium + azimsulfuron POST had lower weed biomass at 45 days after sowing (DAS). At Madhuban, highest grain yield of scented basmati rice (3.43 t ha^−1^) was recorded with the sequential application of pendimethalin PRE fb bispyribac-sodium + azimsulfuron POST. However, at Taraori, yields were similar with pendimethalin or oxadiargyl PRE fb bispyribac-sodium and/or azimsulfuron POST. Applying oxadiargyl by mixing with sand onto flooded field was less effective than spray applications in non-flooded field. The benefit-cost ratio of rice crop was higher with herbicide treatments at both sites as compared with the non-treated weed-free check except single PRE and POST applications and sequential application of oxadiargyl PRE fb oxadiargyl PRE. In a separate experiment conducted at Nagla and Taraori sites, scented rice cultivars' ('CSR 30′ and 'Pusa 1121′) tolerance to three rates of azimsulfuron (15, 25, and 35 g ai ha^−1^) was evaluated over two years (2010 and 2011). CSR 30 (superfine, scented) was more sensitive to higher rates (35 g ai ha^−1^) of azimsulfuron as compared to Pusa 1121 (fine, scented). Crop injuries were 8 and 28% in case of CSR 30; 5 and 15% in Pusa 1121 when applied with azimsulfuron 25 and 35 g ai ha^−1^, respectively. Azimsulfuron applied at 35 g ai ha^−1^ reduced yield in both cultivars but in CSR 30 yield reduction was twofold (11.5%) as that of Pusa 1121 (5.2%).

## Highlights

• Study focus on efficacy and economics of herbicide treatments, and tolerance of scented rice cultivars to azimsulfuron.
• Pendimethalin PRE fb bispyribac-sodium + azimsulfuron POST was most effective herbicide treatment.
• Sandmix application of oxadiargyl (farmers' practice) was less effective compared to spray application.
• Azimsulfuron reduced grain yield of CSR 30 and Pusa 1121 at higher rates.
• Generalized tankmix application of bispyribac + azimsulfuron POST increases total herbicide cost and fetches lower returns.

## Graphical abstract

**Figure:**
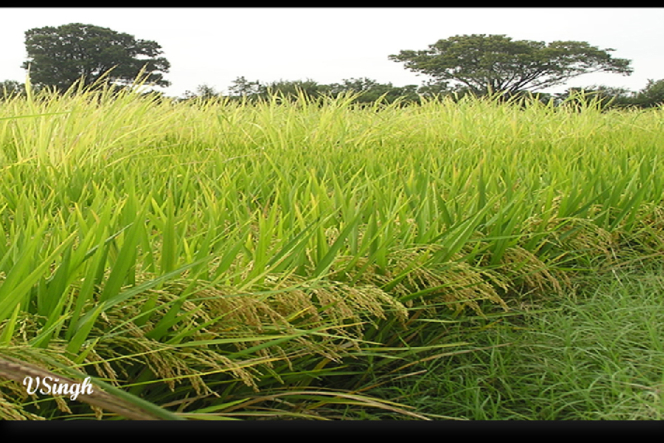

## 1 Introduction

Rice (*Oryza sativa*) is a major cereal crop and staple food for more than half of the world's population. About 90% of the world's rice is produced and consumed in Asia (FAO, 2014). Rice is predominantly grown by transplanting seedlings into puddled (conventional wet-tillage) soil and kept flooded for most part of the growing season. The puddled soil ensures good crop establishment, weed control with standing water, and reduces deep-percolation losses (Sharma et al., 2003). However, the conventional method of rice crop establishment requires a large amount of water, labour, and energy, which are gradually becoming scarce and more expensive. Thus, reducing the profitability and sustainability of puddled transplanted rice (PTR). Because of high rate of withdrawal of ground water in conventional tillage based puddled transplanted rice, water tables in some areas of North-West Indo-Gangetic Plains (IGP) has been declining by 0.1–1.0 m per year, resulting in increased cost of water pumping (Humphreys et al., 2010, Rodell et al., 2009, Hira, 2009). There is evidence that water scarcity prevails in IGP (Tuong et al., 2005) and labour costs have increased dramatically due to migration of rural labour to cities (Chauhan, 2012) as well as other non-agricultural sectors of rural economy. One of the factors behind migration of labour to cities is the implementation of the Mahatma Gandhi National Rural Employment Guarantee Act-2005 which guarantees 100 days of work to all unemployed people in rural India (Anonymous, 2014). Dry direct-seeded rice (DSR) has shown promise under several ecologies and production systems to overcome these challenges, and is considered as potential alternative to PTR.

In DSR systems, dry rice seeds are sown with or without tillage and irrigation is applied periodically to maintain soil at field capacity. DSR has water saving of 11–18% in irrigations (Tabbal et al., 2002) and reduces total labour requirement (11–66%) compared to PTR, depending on season, location, and type of DSR (Kumar et al., 2009, Rashid et al., 2009). Other benefits of DSR include faster and easier planting, improved soil health, higher tolerance to water deficit, less methane emission, and often higher profit in areas with an assured water supply (Datta, 1986, Kumar and Ladha, 2011, Pathak et al., 2009). In addition, DSR matures 7–10 days earlier than the PTR rice allowing timely planting of the succeeding wheat crop (Giri, 1998, Singh et al., 2006).

However, weed management is the major challenge in DSR (Rao et al., 2007, Singh et al., 2007). DSR systems are subject to much higher weed pressure than PTR system (Rao et al., 2007), in which weeds are suppressed by standing water and transplanted rice seedlings, that provide ‘head start’ over germinating weed seedlings (Moody, 1983). In DSR, weeds emerge simultaneously with crop seedlings and grow more quickly in moist soil than in PTR (Khaliq and Matloob, 2011), resulting in severe competition for resources to the crop. Therefore, weeds present the main biological constraint to the success of DSR (Chauhan, 2012), and failure to control weeds result in yield losses ranging from 50 to 90% (Chauhan and Johnson, 2011, Chauhan and Opeña, 2012).

The traditional methods of weed control in rice include hand-weeding by hoe or hand pulling, but this is becoming less common because of labour scarcity at critical time of weeding and increasing labour costs (Chauhan, 2012, Kumar and Ladha, 2011). Moreover, seedlings of some grassy weeds such as *Echinochloa crus-galli* (L.) look similar to rice seedlings (Rao and Moody, 1987, Rao and Moody, 1988), making hand weeding more tedious, difficult, and less effective. However, adoption of DSR technology usually leads to shift in weed flora composition towards difficult-to-control weeds (Singh et al., 2013). In this situation, use of herbicides is becoming more popular in DSR because they are more effective, easy to apply, provide selective control, saves on labour and costs less.

Farmers generally apply herbicides by mixing them in sand for easy operation and prefer to use either single application of PRE or POST herbicides which fails to control diverse weed flora observed in DSR (Chauhan, 2012, Chauhan and Opeña, 2012). However, it is important to use a broad-spectrum herbicide program including PRE and POST herbicides for season-long effective weed control and to avoid shifts toward problematic weed species (Chauhan, 2012, Singh et al., 2008) or evolution of herbicide-resistant weed biotypes. Traditional methods of weed control with manual labour increases the cost of cultivation. Moreover, labour shortage makes it difficult to manage weeds in a timely manner. Return over variable cost with manual weeding is one of the major concerns of farmers in process to adopt DSR in South Asia. Crop safety to new herbicides is another concern particularly in scented rice. Therefore, two studies were conducted to (1) evaluate herbicide options available for effective weed control in DSR, and (2) evaluate tolerance of potential scented rice cultivars (fine and superfine basmati) to azimsulfuron.

## 2 Materials and methods

### 2.1 Experiment 1. Herbicide options for weed management in DSR

#### 2.1.1 Study location

Field studies were conducted at two farmers' participatory research platforms (Madhuban, and Taraori; Karnal, India) of the Cereal Systems Initiative for South Asia (CSISA), a collaborative project of IRRI and CIMMYT, during the *Kharif* (wet) season of 2010. The soil type at both Taraori and Madhuban village was sandy clay loam in texture. Both sites were low in organic matter (0.34–0.37%) with alkaline reaction (pH range of 8.0–8.1). Rice-wheat is the major cropping system of the region and popularly known for basmati/scented rice cultivation.

#### 2.1.2 Experimental design and treatments

Eleven treatments including PRE and POST herbicide combinations (Table 1) were evaluated in a randomized complete block design (RCBD) with three replications at each location. Herbicides included in the study were butachlor (Butaveer^®^, Chambal Fertilizers & Chemicals Ltd.), oxadiargyl (Topstar^®^, Bayer Crop Science), pendimethalin (Stomp^®^, BASF India Ltd.), bispyribac-sodium (Nominee gold^®^, PI Industries), and azimsulfuron (Segment^®^, Dupont India Ltd.).

#### 2.1.3 Experimental details

At each location, a burndown application of glyphosate (1.0 kg ai ha^−1^) was made on the experimental area in mid-May 2010 and was followed by a light tillage with one pass of disc harrow and one pass of spring loaded tyne cultivator followed by planking before seeding. Fungicide-treated (carbendazim@ 0.5 g ai kg^−1^ rice seed) seeds of ‘CSR 30’ cultivar (superfine, scented; basmati cultivar) were planted in the second week of June, 2010, at both the locations. Seeds were drill-seeded at a rate of 20 kg ha^−1^ with a multi-crop seed-cum-fertilizer planter (Dasmesh^®^) at 2–3 cm soil depth. Light irrigation was provided immediately after seeding. All PRE herbicides except oxadiargyl (sand mix application), were sprayed on the third day of irrigation. Oxadiargyl was mixed with sand (8 kg ha^−1^) and broadcast in standing water (2–3 cm) after irrigation on the day of seeding (general farmer practice). All POST herbicides, except sequential PRE application of oxadiargyl, were applied at three-to four-leaf stage of rice [20–22 days after seeding (DAS)]. The sequential oxadiargyl was applied at the two-to three-leaf stage of rice (15 DAS). The herbicides were applied using a battery operated back-pack knapsack sprayer fitted with a flat-fan nozzle and calibrated to deliver 500 L ha^−1^ for PRE spray and 375 L ha^−1^ for POST spray. The area of each plot was 24 m^2^ (6 × 4 m). The crop was managed following the standard recommended practices for the region. Fertilizers, 25 kg N, 30 kg P_2_O_5_, and 25 kg ZnSO_4_ ha^−1^, were applied as a basal dose. N and ZnSO_4_ were broadcasted uniformly and P_2_O_5_ was applied using a multi-crop seed-cum-fertilizer planter while planting. Remaining amount of N (50 kg ha^−1^) was applied in two splits at 40 and 60 DAS. Two sequential foliar sprays of 1% FeSO_4_ were applied at 40 and 47 DAS, though only the Madhuban location showed iron deficiency at this stage. After the first irrigation at the time of seeding, the second light irrigation was applied 5 DAS. Subsequent irrigations were provided at a weekly interval except tillering and panicle emergence stage. Irrigations were applied at 3–4 days interval at tillering stage and during panicle emergence.

Weed biomass and weed density was determined at 20 and 45 DAS from a randomly selected 1 m^2^ quadrat in each plot. Weed samples were oven dried before weighing at 70 °C till the constant weight was achieved. Visual injury (20 and 45 DAS) evaluation for crop was based on chlorosis, and stunting whereas visual weed control included chlorosis, necrosis and plant stand reduction as well. Injury ratings were recorded on a scale of 0–100, where 0 means no injury and 100 means complete death. At harvesting, five rice plant clusters were randomly selected from each treatment to collect data for plant height (cm), panicle length (cm), and number of grains per panicle. Tillers with filled grains were recorded from 1 m^2^ area for each treatment at harvesting. The crop was harvested in the second week of November, 2010, at both sites from two spots (1 × 1 m and 3 × 3 m) area per treatment for accuracy and averaged.

#### 2.1.4 Economics

Net returns for each treatment were calculated over variable cost of production (Table 3). All inputs (pesticides/fertilizers/seeds) for each site were purchased from same source. Some of the operations were same at each site and facilitated by the same contractual-operator with same price. Irrigation was provided with electric motor pump sets with fixed electricity charges. The cost of human labour used for tillage, seeding, irrigation, fertilizer and pesticide application, weeding, harvesting and threshing of crops was based on actual cost to farmers and were estimated considering total acreage and person-hours. Similarly, the time required by a tractor-drawn machine/implement to complete a field operation such as tillage, seeding, and harvesting was recorded. Cost of such field operation was calculated by using time required by such operation, diesel consumed per unit time and market price of diesel. All these costs were summed up to calculate total variable cost of production. Sale price of 'CSR-30′ was averaged over 2 locations to avoid any bias as sale price depends upon open market demand, local timing, and competition. Prices were estimated with average Dollar (US) -Rupee (INR) exchange rate in 2010 ($1 = 45.68).

#### 2.1.5 Statistical analyses

Data were subjected to analysis of variance using JMP Pro v.11 software (SAS, 2013) where herbicide treatments and sites were fixed and blocks at each site were random effect (Table 4). The interaction effect of locations with herbicides was significant and locations varied in weed type and weed pressure, therefore, data were analysed for each site separately, with randomized complete block design (RCBD) with three replications. Weed density data were subjected to square-root √(x + 1) transformation before analysis. Means were separated using Fisher's protected LSD at P ≤ 0.05.

### 2.2 Experiment 2. Evaluating rice cultivars' tolerance to azimsulfuron

Azimsulfuron was commercialized in India in 2012. It was a new herbicide for Indian market then and only few studies had been conducted on azimsulfuron. Since the area under DSR sharply increased in Haryana, India, in 2010, it necessitated the testing of new herbicide molecules for herbicide efficacy and crop safety especially for scented rice which has huge export potential and Haryana is the major exporter of scented (basmati) rice.

#### 2.2.1 Experimental details

Tolerance of two scented rice cultivars ('CSR 30′ and 'Pusa 1121′) to azimsulfuron was evaluated using three herbicide rates (15, 25, and 35 kg ai ha^−1^) applied at 25 DAS. The experiment was conducted at two sites (Nagla and Taraori; Haryana, India) over two years (2010 and 2011) and arranged in a split-plot design with three replications at each site. Cultivars and herbicides were randomized to the main plots and the sub-plots, respectively (Table 2). Seeds were planted in the last week of June in both years. Light irrigation was provided immediately after seeding. Pendimethalin (1000 g ai ha^−1^; PRE) was applied on the 3rd day of irrigation (field capacity) as a standard PRE practice in all treatments and azimsulfuron was applied at 25 DAS. Crop was raised with recommended agronomic practices as explained above for experiment 1. Plots were kept weed-free throughout the season using hand weeding. Visual injury was recorded at 21 d after azimsulfuron application (45 DAS) with reference to non-treated weed free plot for each cultivar in the respective block. Crop was harvested manually in second week of November 2010 and 2011 from two random spots (1 × 1 m and 3 × 3 m) area per treatment for accuracy and averaged, and yield was recorded at 13% moisture.

#### 2.2.2 Statistical analyses

Data were subjected to analysis of variance using SAS (v.9.3). Site-Year and treatments were considered as the fixed effect and blocks nested in site-year, were treated as the random effect. Site-Year and its interaction effects were non-significant (Table 9), therefore data were pooled for both sites (Table 10). Means were separated using Fisher's protected LSD at P ≤ 0.05.

## 3 Results

### 3.1 Herbicide options for weed management in DSR

#### 3.1.1 Weed flora

The experimental plots at Taraori and Madhuban were infested with mixed weed flora. The general ground coverage by weeds (45 DAS) at Taraori was 50% (20% grassy weeds; 20% broadleaved weeds and 10% sedges) whereas, plots at Madhuban were dominated by broadleaved weeds with ground coverage of 50% followed by grassy weeds (15%) and sedges (5%) (Table 2). Because of the variation in weed composition and weed pressure among experimental locations and a significant interaction between site-year and treatments, results are presented separately for each location.

#### 3.1.2 Weed control

##### 3.1.2.1 Madhuban site

Grass weed density at 20 DAS, following PRE application of butachlor, pendimethalin and oxadiargyl were 15, 10–13 and 16–23 plants m^−2^, respectively and were lower compared to 51 plants m^−2^ observed in weedy check (Table 5). Similarly, all PRE herbicide treatments resulted in similar control of broadleaved weeds and sedges. The density of broadleaved weeds following PRE application of butachlor, pendimethalin, and oxadiargyl were 8, 6–7, and 7–8 plants m^−2^, respectively and were 58–68% lower compared to 19 plants m^−2^ in the weedy check at 20 DAS (Table 5). The density of sedges following PRE herbicides were in range of 2–3 plants m^−2^ compared to 7 plants m^−2^ in the weedy check. Biomass (dry weight basis) reductions following PRE herbicide application were identical with all PRE herbicide treatments, with values in the range of 2.4–4 g m^−2^ compared to 10.3 g m^−2^ in weedy check. Following the POST treatments, grass weed density was reduced significantly under all the sequential treatments compared to single application of oxadiargyl PRE and weedy check. The treatment with single application of oxadiargyl PRE or bispyribac-sodium POST had 16 and 11 broadleaved weeds m^−2^, respectively, and showed 27–61% reduction in weed density compared to 64–82% reduction in weed density achieved with the sequential application of PRE fb POST herbicides. Azimsulfuron POST was more effective on broadleaved weeds compared to bispyribac-sodium POST. The density of sedges were in range of 0–1 plants m^−2^ with the sequential applications of PRE fb POST herbicides compared to density of 3, 2, or 7 plants m^−2^ with single PRE or POST-only application. Maximum weed biomass reduction was observed with the sequential application of pendimethalin PRE fb bispyribac-sodium + azimsulfuron POST at 45 DAS (Table 5).

##### 3.1.2.2 Taraori site

The density of grasses, broadleaf weeds and sedges was lower with PRE herbicide treatments compared to weedy check. The density of grass weeds was 12–16, 21–31, and 20 plants m^−2^ following PRE application of pendimethalin, oxadiargyl and butachlor, respectively, compared to 51 plants m^−2^ in the weedy check at 20 DAS (Table 6). The density of broadleaf weeds was reduced by 66–88% with PRE herbicide treatments compared to weedy check. The reduction in weed biomass was in the range of 50–73% with PRE herbicides compared to weedy check. The reduction in density of grasses, broadleaved weeds, and sedges was similar in all the treatments with sequential POST applications of the herbicides and significantly lower densities were observed compared with single PRE application of oxadiargyl and weedy check treatment. The weed biomass was reduced by 67–86% with the sequential application of pendimethlin PRE fb either azimsulfuron or bispyribac-sodium or both together as tankmix application compared to weed biomass reduction of 62–73% with oxadiargyl PRE fb azimsulfuron or bispyribac-sodium at 45 DAS (Table 6).

The results suggest that herbicide treatments were effective in reducing weed density and biomass by more than 75% on an average, and reduced the weed competition for resources and space to the crop. Across the locations, sequential application of herbicides were better compared to single application of either PRE or POST treatments alone in controlling weeds in DSR.

#### 3.1.3 Yield and yield parameters

All the herbicide applications resulted in significantly higher grain yield compared to non-treated control. At Madhuban location, highest grain yield of 3.43 t ha^−1^ was recorded with the sequential application of pendimethalin PRE fb bispyribac-sodium + azimsulfuron POST and it was comparable with the grain yield 3.5 t ha^−1^ obtained in weed-free plots (Table 7). However, at Taraori highest yields of 3.3 and 3.4 t ha^−1^ were obtained with the sequential application of pendimethalin PRE fb either bispyribac-sodium or bispyribac-sodium + azimsulfuron POST, respectively; but none of the herbicide treatments had yield comparable with 3.6 t ha^−1^ obtained in weed-free treatment (Table 7).

Across the locations, higher yields were recorded from sequential application of herbicide treatments compared with single application of oxadiargyl PRE or bispyribac-sodium POST. The yield reduction of 89% and 75% were observed at Madhuban and Taraori respectively, in non-treated control plots compared with weed-free treatments. The treatment of oxadiargyl (sandmix) PRE fb bispyribac-sodium POST had significantly lower yield compared with oxadiargyl PRE fb bispyribac-sodium POST, thus application of oxadiargyl as sandmix had a negative impact on its efficacy.

Maximum numbers of effective tillers (398-446 m^−2^) were observed in the weed free treatment and as expected, minimum numbers of effective tillers (112-162 m^−2^) were recorded in non-treated weedy plots across the locations. The number of effective tillers m^−2^ were 375, 372, 368, and 363 with the sequential application of pendimethalin PRE fb bispyribac + azimsulfuron POST, pendimethalin PRE fb bispyribac-sodium or azimsulfuron POST, and oxadiargyl PRE fb bispyribac-sodium POST compared to 326, 307, and 112 with single application of oxadiargyl PRE, bispyribac-sodium POST and nontreated control, respectively, at Madhuban (Table 7). However, at Taraori, maximum number of tillers m^−2^ were observed with pendimethalin PRE fb either azimsulfuron or bispyribac-sodium or azimsulfuron + bispyribac-sodium POST (Table 8).

The number of grains per panicle was higher by 84–137% and 87–144% with herbicide treatments compared with weedy check at Madhuban and Taraori, respectively. The increase in grains per panicle were more in case of sequential applications of PRE fb POST compared to single PRE or POST. Maximum 1000-grain weight was observed in the weed-free treatment and with herbicide treatments compared to non-treated control. Among herbicide treatments, butachlor PRE or oxadiargyl (sandmix) PRE fb bispyribac-sodium and oxadiargyl POST, had lower 1000- grain weight compared to other herbicide treatments. The panicle length was higher with the weed control treatments compared with weedy check owing to intense weed competition in latter case.

The sequential applications of PRE fb POST herbicides provided better weed control and resulted in higher grain yield compared to single application of either PRE or POST. However, the single application of bispyribac-sodium POST resulted in higher grain yield compared to oxadiargyl PRE at both the locations.

#### 3.1.4 Economics

Across the locations, all weed control treatments provided significantly higher return over variable cost (ROVC) and B:C ratio compared to weedy check. Sequential application of herbicides proved superior to sole application of herbicides either PRE or POST at both the sites. The sequential application of pendimethalin PRE fb bispyribac-sodium + azimsulfuron POST was superior over other herbicide treatments and non-treated weed-free treatment in terms of B:C ratio at both the sites (Table 7, Table 8). At both sites application of bispyribac-sodium after pendimethalin PRE provided significantly higher ROVC and B:C compared to its application after oxadiargyl PRE. Azimsulfuron as POST application performed well compared to bispyribac-sodium in terms of B:C ratio and ROVC owing to higher infestation of broadleaved weeds at Madhuban. Bispyribac-sodium was inferior to azimsulfuron in controlling broadleaved weeds and resulted in lower yield at this location.

At Taroari location, POST application of bispyribac-sodium or azimsulfuron after PRE application of oxadiargyl or butachlor or pendimethalin or tank mix of bispyribac-sodium + azimsulfuron POST provided significantly higher B:C compared to all other treatments.

In general, with availability of these herbicides, farmers have options to control weeds but simultaneously with increasing number of herbicides, application cost of weed control is also increasing.

### 3.2 Evaluating rice cultivars' tolerance to azimsulfuron

Site-year had no effect on rice cultivar tolerance to azimsulfuron application (Table 9). No injury was observed in either of the cultivars with 15 g ai ha^−1^ rate of azimsulfuron. However CSR 30 was found to be more sensitive to higher rates of azimsulfuron as compared to Pusa 1121 (Table 10). Crop injury at 25 and 35 g ai ha^−1^ azimulfuron was 8 and 28% respectively in case of CSR 30 (superfine, scented) cultivar, which was higher compared to 6 and 15%, respectively in Pusa 1121 (fine, scented) cultivar. CSR 30 showed significant yield reduction (3.8%) as herbicide rate increased from 15 to 25 g ai ha^−1^, whereas no yield difference was observed between these rates in case of Pusa 1121. Compared to non-treated weed free treatment, azimsulfuron applied at 35 g ai ha^−1^ reduced yield in both cultivars but yield reduction in CSR 30 was two-folds (11.2%) than that in Pusa 1121 (5.2%). Therefore, higher application rate (>25 g ai ha^−1^) may reduce the yield significantly especially in superfine, scented rice cultivars.

## 4 Discussion

The weed biomass recorded at 20 DAS was similar with all the PRE herbicide treatments (butachlor, pendimethalin and oxadiargyl) and was reduced by 42–77% compared to weedy check. However, pendimethalin applied plots had lesser weed density, particularly grasses, compared with butachlor and oxadiargyl. Butachlor performed better in controlling grasses compared with oxadiargyl. Khaliq and Matloob (2012) also reported that the density of jungle rice reduced to a great extent with PRE application of butachlor and pendimethalin. The effectiveness of pendimethalin PRE in reducing the weed density has been reported by several authors (Moody, 1991, Valverde and Gressel, 2005). The current study has confirmed that the potential herbicide for PRE under DSR in western IGP was pendimethalin with its good grass weed control and activity on some broadleaved weeds. However, it is well documented that without the application of POST herbicides, the rice yield may reduce by 9–60% (McCauley et al., 2005). With sequential application of pendimethalin PRE fb chlorimuron + metsulfuron POST, grassy and broadleaved weed density reduced significantly (Singh et al., 2006). Mahajan et al. (2009) also found that sequential application of pendimethalin (1000 g ha^−1^) PRE fb bispyribac-sodium (30 g ha^−1^) applied 15 DAS provided better control of weeds in DSR. Similarly, Walia et al. (2008) reported pendimethalin 0.75 kg ha^−1^ PRE fb bispyribac-sodium 25 g ha^−1^ POST resulted in 372% increase in rice grain yield compared to weedy check owing to better weed control. The results of the current research are in congruity with previous reports of superior weed control in DSR with PRE application of pendimethlin fb bispyribac-sodium POST (Mahajan and Chauhan, 2013) and pendimethalin PRE fb bispyribac-sodium + chlorimuron + metsulfuron POST (Ganie et al., 2013).

In previous studies, Mahajan and Chauhan (2013) reported lowest weed biomass with the sequential application of pendimethalin PRE fb azimsulfuron POST. For POST application, the tank-mixture of bispyribac-sodium + azimsulfuron would be a potential herbicide combination if both grassy and broadleaved weeds are present in the field. The inclusion of azimsulfuron with bispyribac-sodium for POST application widens the spectrum of weed control because azimsulfuron effectively controls sedges and broadleaved weeds (Singh et al., 2010, Walia et al., 2008). Maximum weed biomass reduction (82–91%) was observed with the sequential application of pendimethalin PRE fb bispyribac-sodium + azimsulfuron POST at 45 DAS. Azimsulfuron effectively controls wide variety of weeds, including broadleaved and sedges like *Cyperus rotundus* and *Dactyloctenium* spp. (Singh et al., 2010, Mahajan and Chauhan, 2013). However, azimsulfuron causes significant injury at higher rates (≥25 g ai ha^−1^) to scented cultivars (scented basmati rice) as evident from current study. Therefore, farmers need to make a balance between desired broadleaved weeds control and corresponding yield reductions if using sensitive and scented cultivars. It was observed that the efficacy of oxadiargyl is reduced with sandmix application compared with spray application. Oxadiargyl (sandmix) application was popular among farmers of the region owing to ease of application and cost effectiveness. However, due to poor weed control, which resulted in lesser yield, economically it fetches less returns by 19–23% compared with oxadiargyl (spray) PRE.

Economically, the sequential application of pendimethalin PRE fb bispyribac-sodium + azimsulfuron POST was superior over non-treated weed-free treatment in terms of B:C ratio (Table 7, Table 8). The grain yield was comparable in these treatments but the cost of labour in maintaining the weed-free situation was higher. Azimsulfuron POST performed better than bispyribac-sodium POST in terms of B:C ratio and ROVC owing to higher infestation of broadleaved weeds at Madhuban. Bispyribac-sodium was inferior to azimsulfuron in controlling broadleaved weeds and resulted in lesser yield at this location.

Higher rice grain yield and economic returns with POST application of bispyribac-sodium was reported earlier by Khaliq et al. (2012). The tankmix application of bispyribac + azimsulfuron increased broad-spectrum weed control (grass and broadleaved) but at the same time also increased total cost of herbicide application. Kumar and Ladha (2011) reported that the increase in net returns with dry-DSR over conventional rice production was US$51 ha^−1^ (averaged across 5 Asian countries) and just US$1 in India. In current study, the net gain in returns in DSR with herbicides over manual weeding was just US$31 (averaged over two locations). The cost of sequential application of herbicides in DSR ranged from approximately US$60 with single PRE and POST to US$90 ha^−1^ for PRE and tankmix POST applications compared to US$175 – US$190 ha^−1^ ($1 = Rs 45.68) in hand weeding in weed-free plots. Moreover, the use of fenoxaprop or other weed specific herbicides either in tankmix with bispyribac-sodium and azimsulfuron (Chauhan et al., 2015, Mahajan and Chauhan, 2015) as early POST or at later stage for effective weed control, would further increase the herbicide application cost. Farmers in state of Haryana and Punjab, India, even use one hand weeding (US$50-US$100 ha^−1^) in addition to sequential herbicides in DSR, for effective weed management (Singh, personal observation). The use of one hand weeding in DSR at 35-60 DAS along with sequential herbicides has also been suggested by many studies (Ahmed and Chauhan, 2014, Chauhan and Abugho, 2013, Ganie et al., 2014). This makes DSR more challenging not only from weed management perspective but also considering economics.

## 5 Conclusion

In dry seeded rice, the herbicide treatments were effective in reducing weed density and biomass by more than 75% on an average. Azimsulfuron POST was more effective on broadleaved weeds compared to bispyribac-sodium POST. Maximum weed biomass reduction was observed with the sequential application of pendimethalin PRE fb bispyribac-sodium + azimsulfuron POST at 45 DAS. Inclusion of azimsulfuron in tankmix with bispyribac-sodium was more efficient at site with higher broadleaved weeds, however, this tankmix application increased total cost also. Therefore, generalized tankmix application of herbicides should be avoided and farmers should be encouraged to apply herbicides based on weed flora. Returns over variable cost will be a driving force for farmers in adopting DSR and achieving better weed control in future. In research studies, an emphasis on economics along with evaluating alternate herbicide options would be more informational to the growers and extension agents.

## Figures and Tables

**Table 1:** List of herbicide options, rate and time of application (Experiment 1).

Treatment no.	Herbicide treatments	Rate	Application time
g ai ha^−1^	(Days after sowing)
1	Butachlor fb bispyribac-sodium	1000 fb 25	3 fb 22
2	Oxadiargyl fb azimsulfuron	90 fb 30	3 fb 22
3	Pendimethlin fb azimsulfuron	1000 fb 30	3 fb 22
4	Oxadiargyl fb bispyribac-sodium	90 fb 25	3 fb 22
5	Pendimethalin fb bispyribac-sodium	1000 fb 25	3 fb 22
6	Oxadiargyl (sandmix) fb bispyribac-sodium	90 fb 25	At planting after irrigation fb 22
7	Oxadiargyl fb oxadiargyl	90 fb 90	3 fb 15
8	Oxadiargyl	90	3
9	Bispyribac sodium	25	22
10	Pendimethalin fb bispyribac-sodium + Azimsulfuron	1000 fb 25 + 22.5	3 fb 22
11	Nontreated control	–	–
12	Weed-free	–	–

Abbreviation: followed by, fb.

**Table 2:** List of weed species at experimental locations, Haryana, India.

Taraori	Madhuban
**Grassy weeds** (20%)	**Grassy weeds** (15%)
*Dactyloctenium aegyptium*	*Echinochloa colona*
*Echinochloa colona*	*Echinochloa crus-galli*
*Echinochloa crus-galli*	*Leptochloa chinensis*
*Leptochloa chinensis*	*Paspalum distichum*
*Paspalum distichum*	
*Cynodon dactylon*	
**Broadleaved** (20%)	**Broadleaved** (50%)
*Ammannia robusta*	*Ammannia robusta*
*Digera arvensis*	*Digera arvensis*
*Lindernia spp.*	*Eclipta prostrata*
*Eclipta prostrata*	*Euphorbia hirta*
	*Phyllanthus niruri*
	*Trianthema portulacastrum*
**Sedges** (10%)	**Sedges** (5%)
*Cyperus difformis*	*Cyperus iria*
*Cyperus rotundus*	*Cyperus rotundus*

*General ground coverage (45 DAS) by weeds in non-treated area at respective locations are given in parentheses ( ).

**Table 3:** Details of variable cost price for farm operations, Karnal, Haryana, India (2010).^a^

Operations/Inputs^b^	Taraori	Madhuban
Cost ($ ha^−1^)	
Tillage	38.3	38.3
Seed cost	24.6	24.6
**PRE herbicides**		
Oxadiargyl	10.4	10.4
Pendimethalin	19.7	19.7
Butachlor	8.2	8.2
**POST herbicides**		
Bispyribac-sodium	30.1	30.1
Azimsulfuron (30 g ai)	31.7	31.7
**Others**		
Hand weeding (for non-treated weed free)	175.1	218.9
Fertilizers	108.3	108.3
Fungicides	27.4	27.4
Insecticides	43.1	43.1
Nipping	27.4	27.4
Permanent labour charges^c^	43.8	65.7
Electricity charges	19.2	19.2
Harvesting cost	109.5	109.5
Post-harvest charges (Market charges/Transportation)	27.4	32.8
Miscellaneous (Tractor operations/Repair etc)	54.7	54.7
Sale price of CSR-30^d^	$ 700 t^−1^	$ 700 t^−1^

a Prices were estimated with average Dollar (US) -Rupee (INR) exchange rate in 2010 ($1 = 45.68).

b All inputs (pesticides/fertilizers/seeds) were from same source; charges (electricity fixed charges) were same for all locations.

c Permanent labour charges were estimated based on total acreage and man-hours.

d Sale price averaged over 3 locations as it depends upon market demand and time of sale.

**Table 4:** Analysis of variance *P* values for the crop injury and yield reduction for experiment 1.

Source of variation	df^a^	Crop injury^b^ (20 DAS)	Crop injury (45 DAS)	Plant height	Tillers	Panicle length	Grains/panicle	Grain weight	Grain yield	Straw yield	Weed biomass (20 DAS)	Weed biomass (45 DAS)
*P*-values										
Location	2 (2)	0.2765	0.0006	<0.0022	0.1618	0.0252	0.0001	<0.1527	<0.0386	<0.0001	0.0011	<0.0001
Herbicide	11 (9)	<0.0001	<0.0001	<0.0001	<0.0001	0.0350	<0.0001	<0.0001	<0.0001	0.0172	<0.0001	<0.0001
Location X Herbicide	22 (18)	<0.0001	<0.0001	0.0069	0.0066	0.0016	0.5470	0.0434	<0.0001	<0.0001	0.9203	<0.0001

a Numbers in parentheses ( ) are df for crop injuries; non-treated weedy and weed free treatments were excluded.

b Crop visual injury was estimated with to reference to non-treated weed free treatment at 20 DAS and 45 DAS; calculated on a scale of 0–100; includes stunting and chlorosis.

**Table 5:** Efficacy of herbicide treatments at 20 DAS and 45 DAS, CSISA research platform, Madhuban, Karnal, India (2010).

Herbicide treatments	Timing	Rate	Weed count; 20 DAS^a^			Weed biomass; 20 DAS	Weed control; 20 DAS^b^	Weed count; 45 DAS^a^			Weed biomass; 45 DAS	Weed control; 45 DAS^b^
Grasses	Broadleaved	Sedges	Grasses	Broad leaved	Sedges
g ai ha^−1^	(No. m^−2^)			(g m^−2^)	(%)	(No. m^−2^)			(g m^−2^)	(%)
Butachlor fb bispyribac-sodium	PRE fb POST	1000 fb 25	3.9 (15)	3.0 (8)	2.0 (3)	3.0	77	2.2 (4)	3.8 (13)	1.4 (1)	38.5	62
Oxadiargyl fb azimsulfuron	PRE fb POST	90 fb 30	4.7 (21)	2.9 (8)	1.7 (2)	3.5	58	2.8 (7)	2.4 (5)	1.1 (0)	31.8	82
Pendimethalin fb azimsulfuron	PRE fb POST	1000 fb 30	3.5 (11)	2.8 (7)	1.6 (2)	2.4	60	2.7 (6)	2.3 (4)	1.0 (0)	30.2	83
Oxadiargyl fb bispyribac-sodium	PRE fb POST	90 fb 25	4.5 (20)	2.9 (8)	2.0 (3)	3.4	78	2.3 (4)	3.2 (9)	1.4 (1)	36.7	68
Pendimethalin fb bispyribac-sodium	PRE fb POST	1000 fb 25	3.7 (13)	2.7 (7)	1.7 (2)	3.5	60	2.1 (3)	3.6 (12)	1.4 (1)	40.4	68
Oxadiargyl (sandmix)^c^ fb bispyribac- sodium	PRE fb POST	90 fb 25	5.9 (33)	3.0 (8)	2.2 (4)	4.0	73	2.7 (6)	3.7 (13)	1.6 (2)	43.0	65
Oxadiargyl fb oxadiargyl	PRE fb PRE	90 fb 90	4.1 (16)	2.8 (7)	1.6 (2)	3.0	37	3.6 (12)	3.5 (11)	1.5 (1)	45.6	67
Oxadiargyl	PRE	90	4.9 (23)	2.9 (8)	1.6 (2)	3.0	60	5.1 (25)	4.1 (16)	1.9 (3)	65.8	52
Bispyribac-sodium	POST	25	7.4 (53)	4.3 (18)	2.8 (7)	7.0	_	2.8 (7)	3.5 (11)	1.6 (2)	52.6	63
Pendimethalin fb bispyribac-sodium + azimsulfuron	PRE fb POST	1000 fb 25 + 22.5	3.3 (10)	2.6 (6)	1.6 (2)	3.0	62	2.0 (3)	2.2 (4)	1.3 (1)	22.5	85
weedy check			7.2 (51)	4.4 (19)	2.8 (7)	10.3	_	7.0 (48)	4.8 (22)	2.9 (7)	127.0	_
weed free			1.0 (0)	1.0 (0)	1.0 (0)	0	_	0	0	0	0	_
LSD_(0.05)_			0.7	0.6	0.3	1.8	8	0.6	0.3	0.3	7.0	7

a Weed density data were subjected to square-root √(x + 1) transformation before analysis and original values of weed emergence are shown in parenthesis.

b Weed control at 20 and 45 DAS includes chlorosis, necrosis, stunting and stand reduction of weedy plants; visual weed control was recorded on a scale of 0–100.

c Oxadiagyl (sandmix) PRE was applied immediately after planting in standing water.

**Table 6:** Efficacy of herbicide treatments at 20 DAS and 45 DAS, CSISA research platform, Taraori, Karnal, India (2010).

Herbicide treatments	Timing	Rate	Weed count; 20 DAS^a^			Weed biomass; 20 DAS	Weed control; 20 DAS^b^	Weed count; 45 DAS^a^			Weed biomass; 45 DAS	Weed control; 45 DAS^b^
Grasses	Broadleaved	Sedges	Grasses	Broadleaved	Sedges
g ai ha^−1^	(No. m^−2^)			(g m^−2^)	(%)	(No. m^−2^)			(g m^−2^)	(%)
Butachlor fb bispyribac-sodium	PRE fb POST	1000 fb 25	4.5 (20)	1.8 (2)	1.5 (1)	3.4	75	1.9 (3)	2.0 (3)	1.7 (2)	20.7	80
Oxadiargyl fb azimsulfuron	PRE fb POST	90 fb 30	4.8 (22)	1.6 (2)	1.6 (2)	3.1	50	2.1 (4)	1.8 (2)	1.7 (2)	17.2	85
Pendimethalin fb azimsulfuron	PRE fb POST	1000 fb 30	4.1 (16)	1.5 (1)	1.5 (1)	2.2	65	2.1 (4)	1.7 (2)	1.3 (1)	15.3	87
Oxadiargyl fb bispyribac-sodium	PRE fb POST	90 fb 25	5.0 (24)	1.8 (2)	1.6 (2)	2.5	52	1.9 (3)	1.8 (2)	1.6 (2)	12.3	78
Pendimethalin fb bispyribac sodium	PRE fb POST	1000 fb 25	3.9 (14)	1.5 (1)	1.5 (1)	2.9	63	1.7 (2)	1.8 (2)	1.4 (1)	10.5	88
Oxadiargyl (sandmix)^c^ fb bispyribac-sodium	PRE fb POST	90 fb 25	5.6 (31)	2.1 (3)	1.7 (2)	4.2	40	2.3 (5)	1.9 (2)	2.1 (3)	24.2	70
Oxadiargyl fb oxadiargyl	PRE fb PRE	90 fb 90	4.6 (21)	1.7 (2)	1.5 (1)	3.0	70	4.0 (15)	2.1 (3)	2.1 (3)	30.3	62
Oxadiargyl	PRE	90 25	4.9 (23)	1.7 (2)	1.7 (2)	3.7	53	4.7 (21)	2.8 (7)	2.1 (3)	42.7	45
Bispyribac-sodium	POST	7.4 (53)	3.3 (10)	2.1 (3)	8.6	_	2.9 (8)	2.3 (5)	1.8 (2)	33.3	60
Pendimethalin fb bispyribac-sodium + azimsulfuron	PRE fb POST	1000 fb 25 + 22.5	3.6 (12)	1.6 (2)	1.4 (1)	2.3	67	1.8 (2)	1.7 (2)	1.6 (2)	7.6	92
Weedy check			7.2 (51)	3.1 (9)	2.3 (4)	8.4	_	7.5 (55)	2.9 (8)	2.6 (6)	82.7	_
Weed free			1.0 (0)	1.0 (0)	1.0 (0)	_	_	1.0 (0)	1.0 (0)	1.0 (0)	0	_
LSD_(0.05)_			0.6	0.4	0.3	0.6	8	0.6	0.4	0.3	7.2	5

a Weed density data were subjected to square-root √(x + 1) transformation before analysis and original values of weed emergence are shown in parenthesis.

b Weed control at 20 and 45 DAS includes chlorosis, necrosis, stunting and stand reduction of weedy plants; visual weed control was recorded on a scale of 0–100.

c Oxadiargyl (sandmix) PRE was applied immediately after planting in standing water.

**Table 7:** Performance of rice ('CSR 30′) in response to herbicide treatments, CSISA research platform, Madhuban, Karnal, India (2010).

Herbicide treatments	Timing	Rate	Crop injury^a^ (%)		Plant height	Panicle length	Tillers	Grains/panicle	1000-grain weight	Grain yield	Benefit-cost^b^	Returns over variable cost
g ai ha^−1^	20 DAS	45 DAS	cm	cm	no. m^−2^	no.	(g)	kg ha^−1^	B:C	($ ha^−1^)
Butachlor fb bispyribac-sodium	PRE fb POST	1000 fb 25	12	8	116	22	343	70	21.3	2800	3.3	1371
Oxadiargyl fb azimsulfuron	PRE fb POST	90 fb 30	0	22	118	22	354	76	21.7	3177	3.7	1620
Pendimethalin fb azimsulfuron	PRE fb POST	1000 fb 30	18	28	120	23	368	75	22.7	3243	3.7	1658
Oxadiargyl fb bispyribac-sodium	PRE fb POST	90 fb 25	0	0	120	23	363	69	22.1	2750	3.3	1334
Pendimethlin fb bispyribac-sodium	PRE fb POST	1000 fb 25	10	0	121	23	372	71	22.0	3053	3.6	1537
Oxadiargyl (sandmix) fb bispyribac-sodium	PRE fb POST	90 fb 25	0	0	118	23	335	66	21.6	2310	2.7	1026
Oxadiargyl fb oxadiargyl	PRE fb PRE	90 fb 90	0	0	119	21	350	68	21.9	2333	3.1	1202
Oxadiargyl	PRE	90	0	0	116	20	307	61	21.5	1487	2.3	734
Bispyribac-sodium	POST	25	–	0	117	19	326	59	22.0	1850	1.8	460
Pendimethalin fb bispyribac-sodium + azimsulfuron	PRE fb POST	1000 fb 25 + 22.5	8	16	119	23	375	74	22.7	3427	3.8	1766
Weedy check			–	–	112	12	112	32	20.9	410	0.5	−264
Weed free			–	–	121	24	398	77	23.0	3463	3.1	1655
LSD_(0.05)_			3	3	2	1	17	8	1.01	176	0.2	123

Abbreviation: fb, followed by; DAS, d after sowing.

a Crop injury was calculated on a scale of 0–100; includes crop stand, stunting and chlorosis.

b Benefit-Cost ratio was calculated over variable cost.

**Table 8:** Performance of rice ('CSR 30′) in response to herbicide treatments, CSISA research platform, Taraori, Karnal, India (2010).

Herbicide treatments	Timing	Rate	Crop injury^a^(%)		Plant height	Panicle length	Tillers	Grains/panicle	1000-grain weight	Grain yield	Benefit-cost^b^	Returns over variable cost
g ai ha^−1^	20 DAS	45 DAS	cm	cm	no. m^−2^	no.	(g)	kg ha^−1^	B:C	($ ha^−1^)
Butachlor fb bispyribac-sodium	PRE fb POST	1000 fb 25	10	3	122	23	346	66	20.7	3040	3.8	1566
Oxadiargyl fb azimsulfuron	PRE fb POST	90 fb 30	0	8	123	25	358	75	21.9	2960	3.6	1496
Pendimethalin fb azimsulfuron	PRE fb POST	1000 fb 30	12	12	124	24	407	73	22.1	3123	3.7	1601
Oxadiargyl fb bispyribac-sodium	PRE fb POST	90 fb 25	0	0	122	25	360	76	21.1	3077	3.8	1590
Pendimethlin fb bispyribac-sodium	PRE fb POST	1000 fb 25	8	0	122	24	398	78	21.3	3310	4.0	1744
Oxadiargyl (sandmix) fb bispyribac-sodium	PRE fb POST	90 fb 25	0	0	119	23	346	74	20.7	2650	3.3	1291
Oxadiargyl fb oxadiargyl	PRE fb PRE	90 fb 90	0	0	120	24	360	70	21.1	2500	3.2	1206
Oxadiargyl	PRE POST	90	0	0	120	22	308	62	21.4	2000	2.6	866
bispyribac-sodium	25	–	0	119	22	324	60	22.1	2300	2.9	1056
Pendimethalin fb bispyribac-sodium + azimsulfuron	PRE fb POST	1000 fb 25 + 22.5	8	20	124	25	408	77	22.1	3393	3.9	1770
Weedy check			–	–	117	17	162	32	20.6	1010	1.4	184
Weed free			–	–	126	27	446	77	22.6	3640	3.6	1850
LSD _(0.05)_			4	3	1	1	28	10	1.1	138	0.2	97

Abbreviation: fb, followed by; DAS, d after sowing.

a Crop injury was calculated on a scale of 0–100; includes crop stand, stunting and chlorosis.

b Benefit-Cost ratio was calculated over variable cost.

**Table 9:** Analysis of variance *P* values for the crop injury and yield reduction for experiment 2 (2010–2011).

Source of variation	df	Crop injury^a^	Yield reduction^b^
*P*-values	
Site-Year (SY)	3	0.1631	0.0622
Variety (V)	1	<0.0001	0.0010
Herbicide rate (H)	2	<0.0001	<0.0001
SY × V	3	0.3300	0.1156
SY × H	6	0.4277	0.0514
V × H	2	<0.001	<0.0001
SY × V × H	6	0.5616	0.5783

a Crop visual injury was estimated with to reference to non-treated weed free treatment at 21 DAT; calculated on a scale of 0–100; includes stunting and chlorosis.

b Site-Year = Each site (Nagla & Taraori) in each year (2010 & 2011); (S x Y = 2 × 2 = 4).

**Table 10:** Rice cultivar tolerance to different rates of azimsulfuron in dry-direct seeded rice, experiment 2 (2010–2011).

Treatment no.	Rice variety	Azimsulfuron rate^a^	Crop injury^b^	Yield reduction^c^	Actual grain yield
(g ai ha^−1^)	(%)	(%)	(Kg ha^−1^)
1	CSR30	15	0	1.5	3610
2	CSR30	25	8	3.8	3525
3	CSR30	35	28	11.5	3245
4	Pusa 1121	15	0	0.9	4638
5	Pusa 1121	25	6	1.0	4632
6	Pusa 1121	35	15	5.2	4451
	LSD_(0.05)_ within variety		3	1.0	
	LSD_(0.05)_ across variety		4	2.0	

a All treatments were sprayed with pendimethalin (1.0 kg ai ha^−1^) PRE at 3 d after sowing as standard practice.

b Crop Injury was recorded visually on a scale of 0–100 at 45 DAS (21 DAT).

c Yield reduction was calculated with reference to non-treated, weed free plots of CSR 30 and Pusa 1121 at respective locations.
